# Allergological work-up in the suspicion of drug hypersensitivity in patients undergoing allergen-specific immunotherapy

**DOI:** 10.1186/1939-4551-8-S1-A72

**Published:** 2015-04-08

**Authors:** Catarina Furlan, Luciana Kase Tanno, Dayane Brandini, Veridiana Pereira-Aun, Wilson Aun, João Ferreira Mello,

**Affiliations:** 1 Hospital Servidor Público Estadual De São Paulo, Brazil

## Background

Poorly documented self-reported drug allergy (DA) is a frequent problem in daily clinical practice and has a considerable impact on prescription choices. Atopy has been described as a risk factor for drug hypersensitivity (DH). The aim of this study was to better investigate the cases of suspicion of DH reported by patients undergoing subcutaneous allergen-specific immunotherapy (SCIT).

## Methods

In this prospective study conducted since 2013, we firstly evaluated the self-reported cases of DH in patients undergoing SCIT in the Allergy Department of our Hospital. The SCIT has been indicated to atopic patients based on the dust mite *in vivo* or *in vitro* specific-IgE (*D.pteronyssinus* and/or *B.tropicalis*) and clinical relevance of these allergens. We excluded cases with unrelated history of DH. For the evaluation of suspected DH, we used the *European Network for Drug Allergy*(ENDA) questionnaire and the DA work-up followed the ENDA recommendations.

## Results

Of all 1400 patients undergoing SCIT evaluated on May/2013, 691(49,3%) replied the first questionnaire, 133(19%) of those self-reported having drug allergies. Forty-five (34%) indicated hypersensitivity to antibiotics(ATB), 46(35%) to non-steroidal anti-inflammatory drugs (NSAIDs), 5(4%) to both ATB and NSAIDs, 31(23%) to other drugs and 6(4%) didn’t remember the medication involved. Of the 133 reports, 65(49%) were evaluated by ENDA questionnaire and 68(51%) refused to go through the drug-allergy evaluation. Of those 65 evaluated cases, forty-two (65%) were women and the mean age was 28 (4 to 70 years). Thirty (46%) cases had history of immediate reaction and the mean time between the reaction and the evaluation was 10 years. Eleven (17%) cases were excluded and the DH investigation has been offered to 54(83%) patients, from whom 38(58%) had possible/probable clinical history of DH. Twenty-five (38%) cases refused or were not interested in undergoing the investigation and 11(17%) are still under investigation. Of 18(28%) who completed the investigation, 11 were with NSAIDs and 7 with antibiotics, all negative.

## Conclusions

The major result of this study confirmed that DH reactions occurred in less than one quarter of patients with a history suggesting possible DA. Negative results on DA work-up may have occurred due to the loss of sensitization and cofactors not included in the diagnostic procedure. Diagnostics tests in individuals with self-reported DA can exclude these conditions.

